# Electroacupuncture Ameliorates Chronic Inflammatory Pain-Related Anxiety by Activating PV Interneurons in the Anterior Cingulate Cortex

**DOI:** 10.3389/fnins.2021.691931

**Published:** 2021-07-05

**Authors:** Fangbing Shao, Junfan Fang, Mengting Qiu, Sisi Wang, Danning Xi, Xiaomei Shao, Xiaofen He, Jianqiao Fang, Junying Du

**Affiliations:** Department of Neurobiology and Acupuncture Research, The Third School of Clinical Medicine, Zhejiang Chinese Medical University, Key Laboratory of Acupuncture and Neurology of Zhejiang Province, Hangzhou, China

**Keywords:** electroacupuncture, chronic inflammatory pain, anterior cingulate cortex, parvalbumin, anxiety

## Abstract

Chronic inflammatory pain is a common clinical disease that tends to be associated with negative emotions such as anxiety and depression. The anterior cingulate cortex (ACC) is involved in pain and pain-related anxiety, and γ-aminobutyric acid (GABA)-ergic interneurons play an important role in chronic pain and anxiety. Electroacupuncture (EA) has good analgesic and antianxiety effect, but the underlying mechanisms have not yet been fully elucidated. In this study, we established a chronic inflammatory pain model and observed that this model induced anxiety-like behaviors and decreased the numbers of parvalbumin (PV) and somatostatin (SOM) positive cells. Activation of PV but not SOM interneurons by chemogenetic techniques alleviated anxiety-like behaviors and pain sensation. EA treatment improved pain sensation, anxiety-like behaviors and increased the number of PV- positive cells in the ACC, but did not affect on the number of SOM-positive cells in the ACC. Moreover, specific inhibition of PV interneurons by chemogenetic methods reversed the analgesic and antianxiety effects of EA. These results suggest that EA ameliorates chronic inflammatory pain and pain-related anxiety by upregulating PV but not SOM interneurons in the ACC.

## Introduction

Chronic pain not only causes painful physical sensations but also often accompanies adverse emotional reactions such as depression and anxiety (Xie et al., 2012; Williams and Craig, 2016). It has been reported that more than 50% of chronic pain patients suffer from anxiety disorder (Feingold et al., 2017), and this negative emotion can also aggravate the feeling of pain (Villemure and Bushnell, 2009). Chronic inflammatory pain is a type of chronic pain that is often associated with depression and anxiety comorbidities (Harth and Nielson, 2019). Conventional combination treatments of analgesic, anti-inflammatory and antianxiety drugs cannot meet the medical need for treating chronic inflammatory pain because of their various side effects or the development of tolerance when they are applied for long periods of time (Breivik et al., 2006).

Electroacupuncture (EA) is a treatment method that combines traditional acupuncture with electrical nerve stimulation based on the basic theory of traditional Chinese medicine. Both animal and human studies have shown that EA can significantly improve chronic inflammatory pain and pain-induced disorders (Lin et al., 2020). Our previous studies have demonstrated that EA relieved chronic inflammatory pain and pain-related anxiety by downregulating PKMzeta expression and upregulating the neuropeptide S (NPS)/NPSR system in the anterior cingulate cortex (ACC) (Du et al., 2017; Du et al., 2020; Xu et al., 2020). However, the underlying mechanisms by which EA ameliorates chronic inflammatory pain-related anxiety has not yet been fully elucidated.

It is well known that dysfunction of central γ-aminobutyric acid (GABA)-ergic interneurons is associated with anxiety and depression (Möhler, 2012). Recently, GABAergic interneurons in the ACC and basolateral amygdala (BLA) were also shown to be involved in chronic inflammatory and neuropathic pain (Koga et al., 2018; Yue J. et al., 2018; Gungor and Johansen, 2019). Another paper reported that GABAergic cell transplantation into the ACC reduced neuropathic pain aversiveness (Juarez-Salinas et al., 2019). Therefore, GABAergic interneurons in the ACC may play an important role in pain-related emotion. GABAergic interneurons have a high degree of heterogeneity and have been classified by morphological, electrophysiological and specific molecular markers (DeFelipe et al., 2013; Ferguson and Gao, 2018). The subtypes of GABAergic interneurons mainly include parvalbumin (PV), somatostatin (SOM), and vasoactive intestinal peptide (VIP), neuropeptide Y (NPY) (Tremblay et al., 2016). It has been reported that activation of PV but not SOM interneurons in the ACC had analgesic effects on inflammatory pain (Kang et al., 2015), and reduction of PV but not SOM interneurons in the hippocampus led to anxiety or depression-like behavior (Wang et al., 2021).

In this study, we aimed to investigate whether PV or SOM interneurons in the ACC contribute to anxiety-like behavior induced by chronic inflammatory pain and whether EA modulates chronic inflammatory pain-related anxiety-like behaviors through these interneurons. To test this hypothesis, we established a chronic inflammatory pain rat model with Complete Freund’s Adjuvant (CFA) and employed behavioral tests (pain and anxiety-like behaviors) and immunohistochemical and chemogenetic techniques to determine the underlying mechanisms of EA in the treatment of chronic inflammatory pain-related anxiety.

## Materials and Methods

### Animals

Adult healthy male Sprague-Dawley (SD) rats (180–240 g, purchased from the Shanghai Laboratory Animal Center) were group housed with a maximum of 4 animals in individual cages under controlled conditions (temperature: 23–25°C; humidity: 40–60%; 12:12 h light/dark cycle) and given food and water *ad libitum*. All experiments were conducted at 9:00–17:00 each day.

### Chronic Inflammatory Pain Model

A chronic inflammatory pain model was established by subcutaneously injecting 0.1 mL CFA (Sigma, United States) into the left hind paw of the rats. Rats in the control group were injected with the same volume of sterile 0.9% saline.

### Virus Injection

All rats were acclimatized to the laboratory environment at least 7 days before surgery. A stereotactic frame (RWD, 68025, China) was used for craniotomy under deep anesthesia with 2% isoflurane. Skulls were fully exposed to locate bregma and lambda and drilled using a dental drill (WPI, OmniDrill35, United States) at the target location. A 10-μL WPI nanofill syringe was connected to a microsyringe pump controller (WPI, UMP3-MICRO4, United States). For chemogenetic manipulations, rAAV-fPV-CRE-bGH PA (titer: 2.64 × 10^12^ vg/mL) and rAAV-Ef1α-DIO-hM3D(Gq)-mCherry-WPRE (titer: 5.72 × 10^12^ vg/mL) were premixed at a ratio of 1:1 for the specific activation of PV interneurons; rAAV-fPV-CRE-bGH PA (titer: 2.64 × 10^12^ vg/mL) and rAAV-Ef1α-DIO-hM4D(Gi)-mCherry-WPREs (titer: 5.63 × 10^12^ vg/mL) were premixed at a 1:1 ratio for the specific inhibition of PV interneurons; rAAV-fSST-CRE-bGH PA (titer: 2.19 × 10^12^ vg/mL) and rAAV-Ef1α-DIO-hM3D(Gq)-mCherry-WPRE (titer: 5.72 × 10^12^ vg/mL) were premixed at a ratio of 1:1 for the specific activation of SOM interneurons; and rAAV-PV-mCherry-pA (titer: 5.10 × 10^12^ vg/mL) and rAAV-SST-mCherry-pA (titer: 2.70 × 10^12^ vg/mL) were control viruses. A 400 nL volume of virus was injected into the ACC (AP: + 2.76 mm, ML: ±0.75 mm, DV (from the brain): +1.4 mm) at a rate of 50 nL/min. The activating virus was injected on the right side, and the inhibitory viruses were injected on both sides. The model was established at 7 days after virus injection. Five weeks after the virus injection, behavioral tests were measured 30 min after administration of Clozapine N-oxide (CNO i.p., 2 mg/kg; Wuhan BrainTVA, China). All viruses used in this study were provided by Wuhan BrainTVA Co., Ltd.

### Behavioral Tests

Mechanical hypersensitivity in rats was determined using paw withdrawal thresholds (PWTs). Anxiety-like behaviors in rats were determined using the open field (OF), elevated zero maze (EZM), and novelty-suppressed feeding (NSF) tests. Moreover, we only performed one behavioral test 1 day by the same operator during 9:00–17:00.

#### PWTs

Paw withdrawal thresholds was measured with a series of von Frey hairs (0.4, 0.6, 1.0, 2.0, 4.0, 6.0, 8.0, 15.0, and 26.0 g; North Coast, United States) using the up-down method (Chaplan et al., 1994). Rats were placed on an elevated wire mesh screen and covered with transparent boxes. After 30 min of habituation, von Frey hairs were placed onto the plantar surface for 6–8 s until the rat removed or licked its paw. The initial hair strength was 4 g; the hair strength was increased each trial until a positive response appeared, which was marked as “X,” then the hair strength was reduced each trial until there was no response, which was marked as an “O.” The first hair strength where the symbols “OX” or “XO” crossed were used as a starting point and tested four consecutive times using a series of “O” and “X” combinations. The results were calculated by the following formula: PWTs (g) = 10 ^∧^ (xf + k × δ-4). “xf” was the logarithmic value of the von Frey hair strength last used, “k” was the corresponding value of the resulting sequence in the k-value table, and “δ” was the mean difference between stimuli (here, 0.231). If the result was greater than 26 g or less than 0.4 g, it was recorded as 26 or 0.4 g.

#### OF

The OF test is one of the most commonly used tests of anxiety behavior because it triggers a conflict between an animal’s desire to explore and its fear of venturing into the OF (Sarkar, 2020). After 30 min of habituation, a rat was put into the central area of the box (100 cm × 100 cm × 50 cm) in a dimly lit room. A video camera was used to record the motor behavior of rats in the OF for 5 min. The time spent in the central area, percentage of distance traveled in the central area and total distance traveled were calculated by Smart 3.0 software (Panlab, United States).

#### EZM

The EZM test is an improved version of the elevated plus maze and involves the conflict between an animal wanting to explore new areas, and not wanting to leave the protected closed arm (Campos et al., 2013). The circular device consists of two elevated open arms and closed arms (100 cm × 50 cm × 25 cm). After 30 min of habituation, a rat was placed between an open arm and a closed arm, facing the open arm in a dimly lit room. A video camera was used to record the motor behavior of rats in the EZM for 5 min. The percentage of distance traveled in the open arm and time spent in the open arm were calculated by Smart 3.0 software (Panlab, United States).

#### NSF

The NSF test is often used to assess anxiety-like behavior because it triggers a conflict between an animal’s urge to feed and its fear of venturing into the center of the box in a bright new environment (Yalcin et al., 2011). We prepared a homemade black box (40 cm × 40 cm × 30 cm) with 1 cm of corn cob padding, and a food was placed on paper in the middle of the box. All rats were deprived of food but not water for 24 h. At the time of testing, a rat was placed in a corner of the box, and the latency to initiate feeding was recorded with a maximum of 5 min. The tested rat was immediately placed alone in a home cage, after the rat began to eat the food, with weighed food for 5 min. Subsequently, food consumption of the tested rat was recorded. If the rats did not begin feeding within 5 min, it was recorded as 5 min, and then the food consumption test was conducted.

### EA Treatment

The EA intervention was conducted from day 26 to 31. The rats in the EA group were fixed and locally disinfected. Acupuncture needles (specification 0.25 mm × 13 mm) were inserted at bilateral Zusanli (ST36) and Kunlun (BL60) acupoints to a depth of 5 mm, and then the HANS–200A Acupoint Nerve Stimulator (Huawei Co., Ltd., China) connection was made at the bilateral ST36 and BL60. The EA stimulation parameters were as follows: 2/100 Hz, 0.5–1.5 mA (stimulus intensity was initially 0.5 mA and increased by 0.5 mA every 10 min of treatment). Shallow punctures into the subcutaneous acupoints without current delivery were administered to rats in the sham EA group. All rats were prepared with the same fixation method.

In the chemogenetic experiments, CNO was intraperitoneally injected first, followed by the EA intervention for 30 min.

### Immunofluorescence

Rats under deep anesthesia with pentobarbital (80 mg/kg, i.p.) were transcardially perfused with 4°C saline followed by 4% paraformaldehyde in 0.1 M PBS (Solarbio, China) after the last behavioral test. It is worth noting that the rats had to be killed within 2 h after CNO injection. Brains were harvested and post-fixed for 24 h in 4% paraformaldehyde at 4°C, followed by sequential dehydration in 15 and 30% sucrose. OCT-embedded brains were sectioned into 30-μm-thick sections on a cryostat microtome NX50 (Thermo, United States). Sections were rinsed in TBST four times and incubated for 1 h at 37°C with 10% donkey serum (0.3% Triton X-100/TBST buffer). The sections were incubated overnight at 4°C with the following antibodies: rabbit anti-PV (1:400, ab11427, Abcam, United States), rabbit anti-SOM (1:400, GTX133119, Gene Tex, United States), rabbit anti-VIP (1:200, ab272726, Abcam, United States), and rabbit anti-NPY (1:800, ab10980, Abcam, United States). Subsequently, the sections were washed six times in TBST and incubated with pre-adsorbed secondary donkey anti-rabbit IgG H&L (Alexa Fluor^®^ 488) (1:500, ab150061, Abcam, United States) for 1 h at 37°C. The sections were washed with TBST six times. Then, the sections were incubated with diamidino-phenyl-indole (DAPI). When dry, the sections were cover-slipped. Images were scanned using the Imager M2 microscope (ZEISS, Germany). Quantitative analyses of the number of positive cells in the ACC were performed using ImageJ software.

### Statistical Analysis

The results are presented as the mean ± standard error of the mean (SEM) and were analyzed by SPSS 20.0. Repeated measures analysis of variance (ANOVA) was used to analyze the PWTs followed by Bonferroni’s *post hoc* tests. Comparisons of data from two groups were analyzed using an unpaired Student’s *t* test. Comparisons of data from multiple groups were analyzed using one-way ANOVA, for comparisons between two groups, Bonferroni tests were used when variances were homogeneous, and Dunnett’s T3 tests were used when variances were not homogeneous. *P* < 0.05 was used as the criterion for statistical significance.

## Results

### Chronic Inflammatory Pain Induced Anxiety-Like Behavior Changes in Rats

**FIGURE 1:**
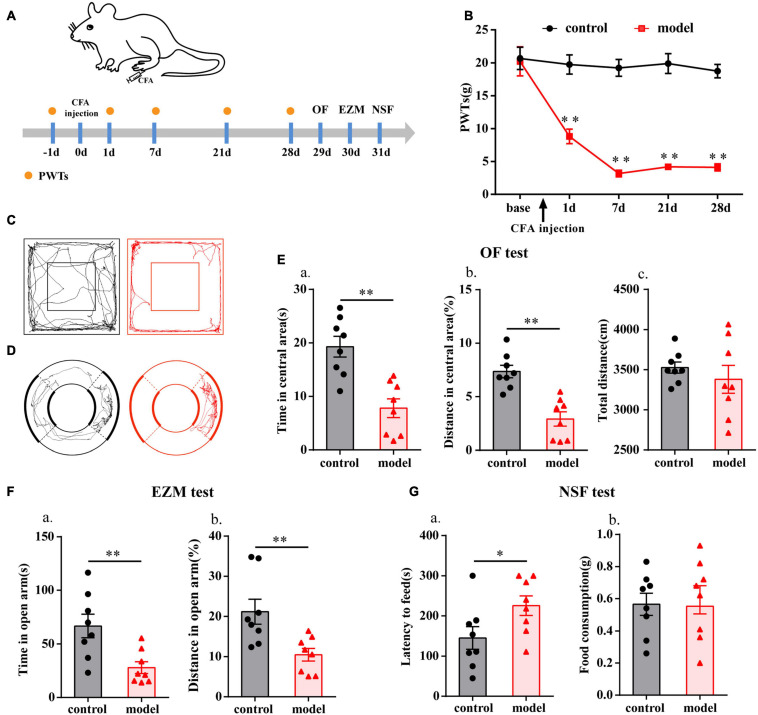
Chronic inflammatory pain induced anxiety-like behavior changes in rats. **(A)** Schematic of the experimental timeline. **(B)** PWTs of rats in response to von Frey hairs. **(C)** The trajectories of rats in the OF on day 29. **(D)** The trajectories of rats in the EZM on day 30. **(E)** Quantification of behavioral parameters in the OF test. **(a)** The time spent in the central area, **(b)** the percentage of distance traveled in the central area, and **(c)** the total distance traveled throughout the arena. **(F)** Quantification of behavioral parameters in the EZM test. **(a)** The time spent in the open arm, and **(b)** the percentage of distance traveled in the open arm. **(G)** Quantification of behavioral parameters in the NSF test. **(a)** The latency to initiate feeding and **(b)** food consumption. All data represent the mean ± SEM, *n* = 8/group, ^∗^*P* < 0.05 and ^∗∗^*P* < 0.01 compared to the control group.

The flow chart of the experimental design is shown in Figure 1A. The baseline PWTs in the two groups before CFA injection were similar (*P* > 0.05; Figure 1B), but the ipsilateral PWTs of the model group were significantly decreased on days 1, 7, 21, and 28 after CFA injection (*P* < 0.01; Figure 1B).

Twenty-eight days after CFA injection, compared with control group, the model group rats displayed multiple anxiety-like behaviors, including behavior in the OF test (i.e., decreased time in central area and percentage of distance in central area, *P* < 0.01; Figures 1C,E), EZM test (i.e., decreased percentage of distance in open arm and time in open arm, *P* < 0.01; Figures 1D,F), and NSF test (i.e., increased feeding latency, *P* < 0.05; Figure 1G). To check whether there was any influence on general behavioral activity after CFA injection, we compared the total distance traveled by the two groups of rats in the OF test and found that there was no difference (*P* > 0.05; Figure 1Ec).

### PV Interneurons and SOM Interneurons Decreased With Chronic Inflammatory Pain

**FIGURE 2:**
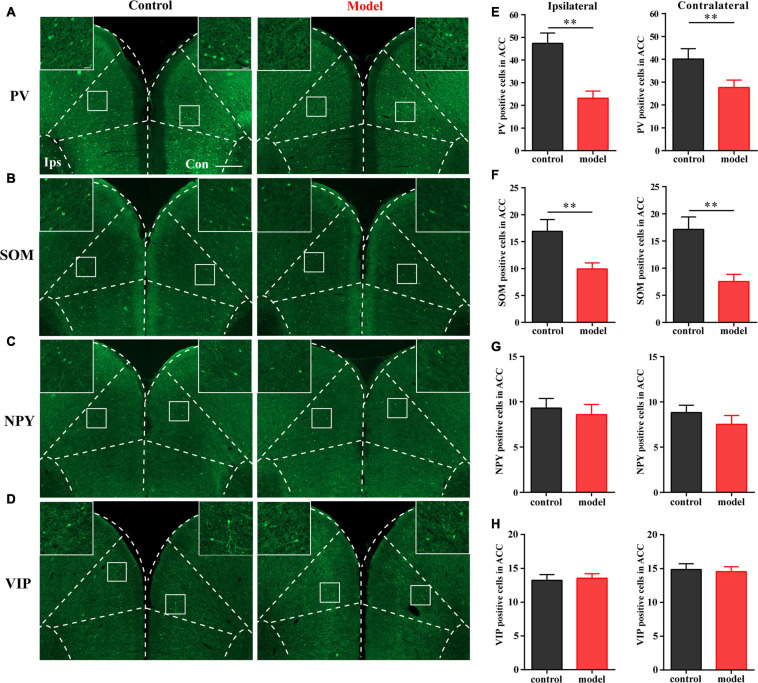
Parvalbumin (PV) interneurons and SOM interneurons were decreased with chronic inflammatory pain. Representative images of PV **(A)**, SOM **(B)**, NPY **(C)**, and VIP **(D)** positive cells in the bilateral ACC in the control and model groups of rats. Quantification of the immunofluorescence results of PV **(E)**, SOM **(F)**, NPY **(G)**, and VIP positive **(H)** cells in the ipsilateral and contralateral ACC in the control and model groups (whole figure scale bars: 500 μm; local figure scale bars: 50 μm). All data represent the mean ± SEM, *n* = 3/group. ^∗∗^*P* < 0.01 compared to the control group.

As shown in Figure 2, the numbers of PV- and SOM-positive cells were reduced in the bilateral ACC in the model group compared with those in the control group (*P* < 0.01; Figures 2A,B,E,F). There was no significant difference in the number of VIP- and NPY-positive cells between the two groups (*P* > 0.05; Figures 2C,D,G,H). These results indicated that PV and SOM interneurons may be associated with chronic inflammatory pain and pain-related anxiety-like behavior.

### Chemogenetic Activation of PV Interneurons Alleviated Anxiety-Like Behavior in Rats With Chronic Inflammatory Pain

**FIGURE 3:**
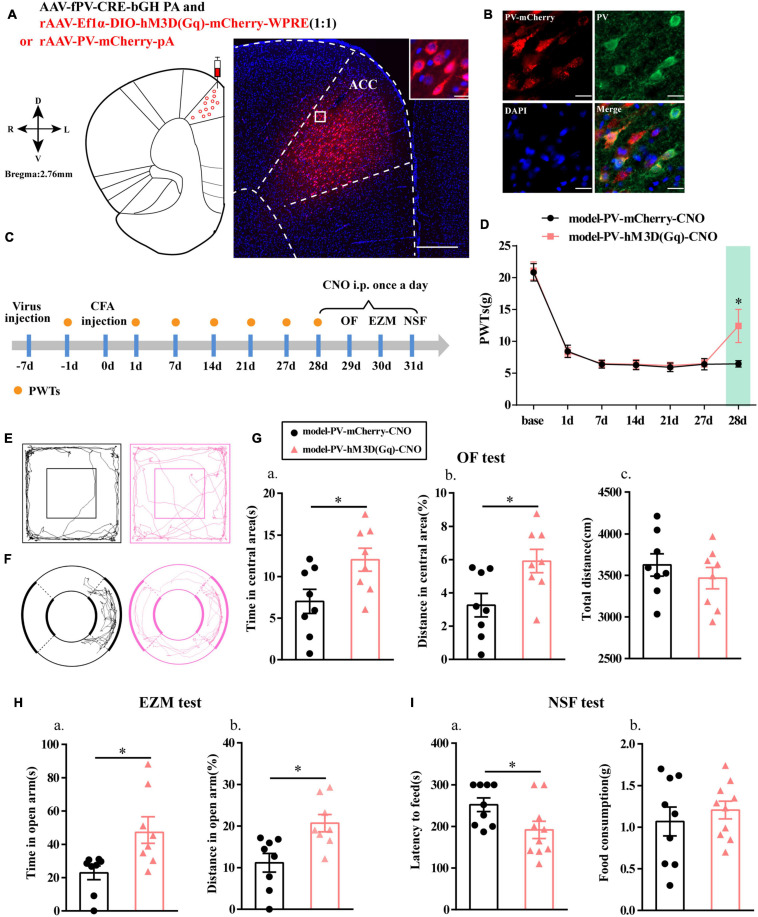
Chemogenetic activation of PV interneurons alleviated anxiety-like behavior in rats with chronic inflammatory pain. **(A)** A representative figure showing the expression of mCherry in the ACC (whole figure scale bars: 500 μm; local figure scale bars: 20 μm). **(B)** Representative images of PV interneurons (green) merged with mCherry (red) in the ACC in the model rats (scale bars: 20 μm). **(C)** A schematic of the experimental design. **(D)** PWTs changes with chemogenetic activation of PV neurons in the ACC (*n* = 11 in the model-PV-mCherry-CNO group; *n* = 12 in the model-PV-hM3D(Gq)-CNO group). **(E)** The trajectories of rats in the OF. **(F)** The trajectories of rats in the EZM. **(G)** Quantification of behavioral parameters in the OF test (*n* = 8 in the model-PV-mCherry-CNO group; *n* = 8 in the model-PV-hM3D(Gq)-CNO group). **(a)** The time spent in the central area, **(b)** the percentage of distance traveled in the central area, and **(c)** the total distance traveled throughout the arena. **(H)** Quantification of behavioral parameters in the EZM test (*n* = 8 in the model-PV-mCherry-CNO group; *n* = 8 in the model-PV-hM3D(Gq)-CNO group). **(a)** The time spent in the open arm and **(b)** the percentage of distance traveled in the open arm **(I)** Quantification of behavioral parameters in the NSF test (*n* = 9 in the model-PV-mCherry-CNO group; *n* = 10 in the model-PV-hM3D(Gq)-CNO group). **(a)** The latency to initiate feeding and **(b)** food consumption. All data represent the mean ± SEM; ^∗^*P* < 0.05 compared to the model-PV-mCherry-CNO group.

To further clarify the function of PV interneurons in chronic inflammatory pain and pain-related anxiety-like behavior, we specifically activated PV interneurons by chemogenetic method. The flow chart of the experimental design is shown in Figure 3C. Before this, we first examined the specificity of the virus, and the immunofluorescence results showed that cells with mCherry signals were observed in the ACC and had a high co-localization with PV-positive cells (Figures 3A,B).

Paw withdrawal thresholds for the rats in both groups were significantly decreased at 1, 7, 14, 21, and 27 days after CFA injection, and the PWTs in the model-PV-hM3D(Gq)-CNO group were significantly higher than those in the model-PV-mCherry-CNO group after CNO administration (*P* < 0.05; Figure 3D).

Rats in the model-PV-hM3D(Gq)-CNO group exhibited an increase in the time and percentage of distance in central area relative to the model-PV-mCherry-CNO group in the OF test (*P* < 0.05; Figures 3E,G). In addition, the model-PV-hM3D(Gq)-CNO rats spent more time in open arm in the EZM test and a greater percentage of distance in open arm than the model-PV-mCherry-CNO rats (*P* < 0.05; Figures 3F,H). Furthermore, the feeding latency for the model-PV-hM3D(Gq)-CNO group was significantly shorter in the NSF test (*P* < 0.05; Figure 3I). Importantly, the hM3D(Gq) manipulation had no effect on locomotor activity (*P* > 0.05; Figure 3Gc). These results suggested that activation of PV interneurons not only relieved chronic inflammatory pain sensation but also ameliorated chronic inflammatory pain-induced anxiety in the model rats.

### Chemogenetic Activation of SOM Interneurons Had No Effect on Anxiety-Like Behavior in Rats With Chronic Inflammatory Pain

**FIGURE 4:**
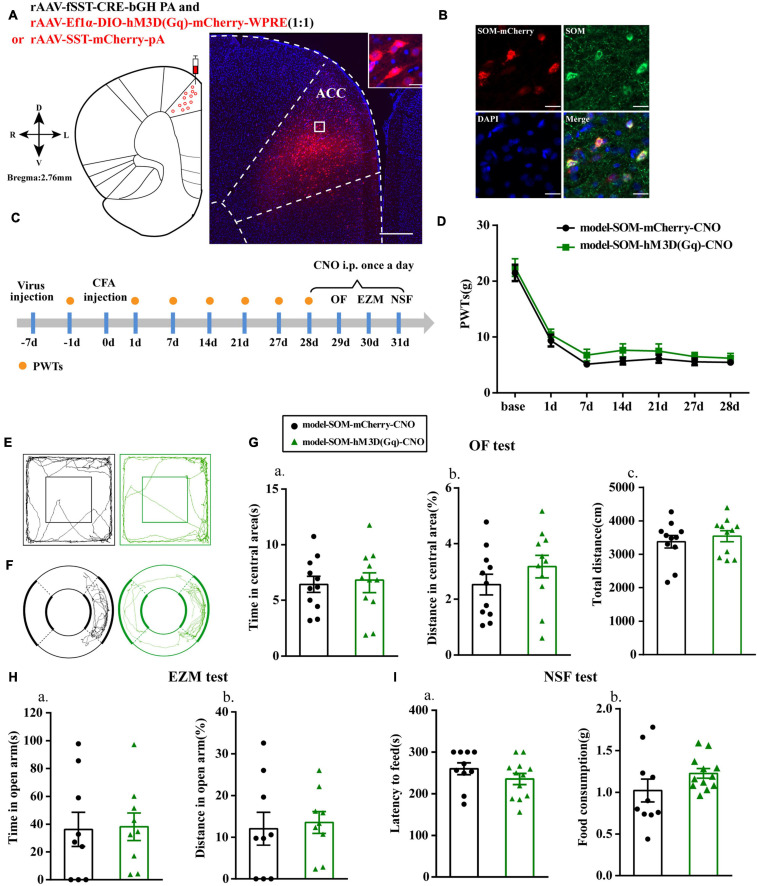
Chemogenetic activation of SOM interneurons had no effect on anxiety-like behavior in rats with chronic inflammatory pain. **(A)** A representative figure showing the expression of mCherry in the ACC (whole figure scale bars: 500 μm; local figure scale bars: 20 μm). **(B)** Representative images of SOM interneurons (green) merged with mCherry (red) in the ACC in the model rats (scale bars: 20 μm). **(C)** A schematic of the experimental design. **(D)** PWTs changes with chemogenetic activation of SOM interneurons in the ACC (*n* = 12 in the model-SOM-mCherry-CNO group; *n* = 12 in the model-SOM-hM3D(Gq)-CNO group). **(E)** The trajectories of rats in the OF. **(F)** The trajectories of rats in the EZM. **(G)** Quantification of behavioral parameters in the OF test (*n* = 11 in the model-SOM-mCherry-CNO group; *n* = 11 in the model-SOM-hM3D(Gq)-CNO group). **(a)** The time spent in the central area, **(b)** the percentage of distance traveled in the central area, and **(c)** the total distance traveled throughout the arena. **(H)** Quantification of behavioral parameters in the EZM test (*n* = 9 in the model-SOM-mCherry-CNO group; *n* = 9 in the model-SOM-hM3D(Gq)-CNO group). **(a)** The time spent in the open arm, **(b)** the percentage of distance traveled in the open arm. **(I)** Quantification of behavioral parameters in the NSF test (*n* = 10 in the model-SOM-mCherry-CNO group; *n* = 12 in the model-SOM-hM3D(Gq)-CNO group). **(a)** The latency to initiate feeding and **(b)** the food consumption. All data represent the mean ± SEM.

We then specifically activated SOM interneurons to observe their effects on pain-related anxiety-like behaviors based on the fluorescence results. Before this, we first examined the specificity of the virus, and the immunofluorescence results showed that cells with mCherry signals could be observed in the ACC and had high co-localization with SOM-positive cells (Figures 4A,B). PWTs of rats in both groups were significantly decreased at 1, 7, 14, 21, and 27 days after CFA injection. Unlike the effects observed after activation of PV interneurons, the PWTs in the model-SOM-hM3D(Gq)-CNO group were not significantly increased after CNO administration compared with those in the model-SOM-mCherry-CNO group (*P* > 0.05; Figure 4D), and activation of SOM interneurons did not affect anxiety-like behaviors in the OF, EZM, and NSF tests, as shown in Figure 4. These results suggested that SOM interneurons are not associated with chronic inflammatory pain or pain-related anxiety.

### EA Effectively Reduced Anxiety-Like Behaviors in Rats With Chronic Inflammatory Pain

**FIGURE 5:**
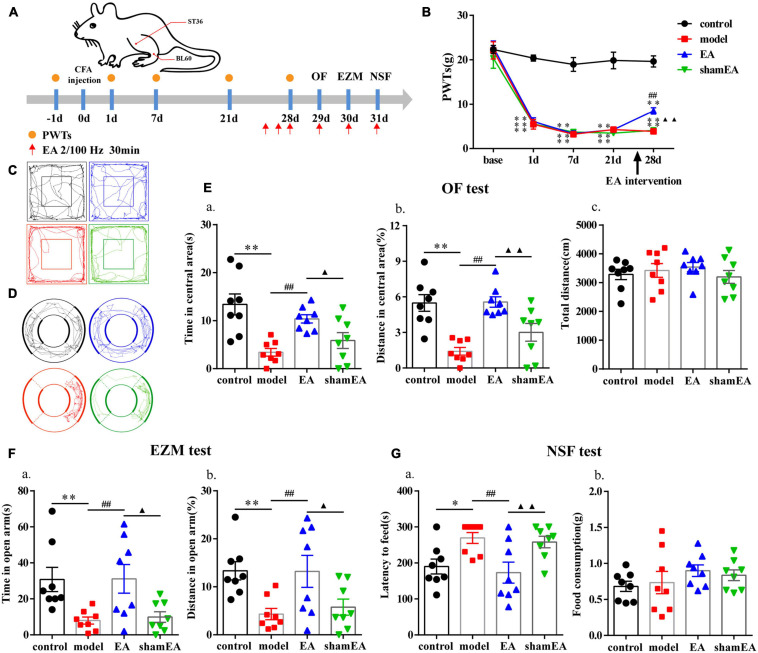
Electroacupuncture (EA) effectively reduced anxiety-like behaviors in rats with chronic inflammatory pain. **(A)** The procedure for the EA stimulation experiment and a schematic picture of the locations of acupoints ST36 and BL60 in rats. **(B)** The analgesic effects of EA stimulation in the model rats. **(C)** The trajectories of rats in the different groups in the OF. **(D)** The trajectories of rats in the different groups in the EZM. **(E)** Quantification of behavioral parameters in the OF test. **(a)** The time spent in the central zone, **(b)** the percentage of distance traveled in the central zone, and **(c)** the total distance traveled throughout the arena. **(F)** Quantification of behavioral parameters in the EZM test. **(a)** The time spent in the open arm and **(b)** the percentage of distance traveled in the open arm **(G)** Quantification of behavioral parameters in the NSF test. **(a)** The latency to initiate feeding and **(b)** food consumption. All data represent the mean ± SEM, *n* = 8/group. ^∗^*P* < 0.05 and ^∗∗^*P* < 0.01, control group vs. model group; ^##^*P* < 0.01, EA group vs. model group; ^▲^*P* < 0.05 and ^▲▲^*P* < 0.01, EA group vs. shamEA group.

The flow chart of the experimental design is shown in Figure 5A. The ipsilateral PWTs of rats were dynamically detected before CFA injection and 1, 7, and 21 days after CFA injection. Before the CFA injection, there was no significant difference in PWTs among all groups (*P* > 0.05; Figure 5B). The PWTs in the CFA-injected rats significantly decreased compared with those in the control rats from 1 to 21 days after CFA injection (*P* < 0.01; Figure 5B). The EA intervention was started at 26 days after CFA injection. Compared with the model group, the EA group had PWTs that significantly increased (*P* < 0.01; Figure 5B) after continuous intervention for 3 days. After 4–6 days of the EA intervention, we assessed the anxiety-like behaviors of the rats in the OF, EZM, and NSF tests. EA treatment increased the time and percentage of distance spent in central area of the OF test (*P* < 0.01; Figures 5C,E) and their percentage of distance and time spent in open arm in the EZM test (*P* < 0.01; Figures 5D,F). In addition, EA animals also had lower feeding latency in the NFS test than the model animals (*P* < 0.01; Figure 5G). The anxiety-like behaviors of rats in the shamEA group did not significantly change compared with those in the model group. These results demonstrated that EA had analgesic and antianxiety effects.

### EA Treatment Increased the Number of PV Interneurons in the Chronic Inflammatory Pain Model

**FIGURE 6:**
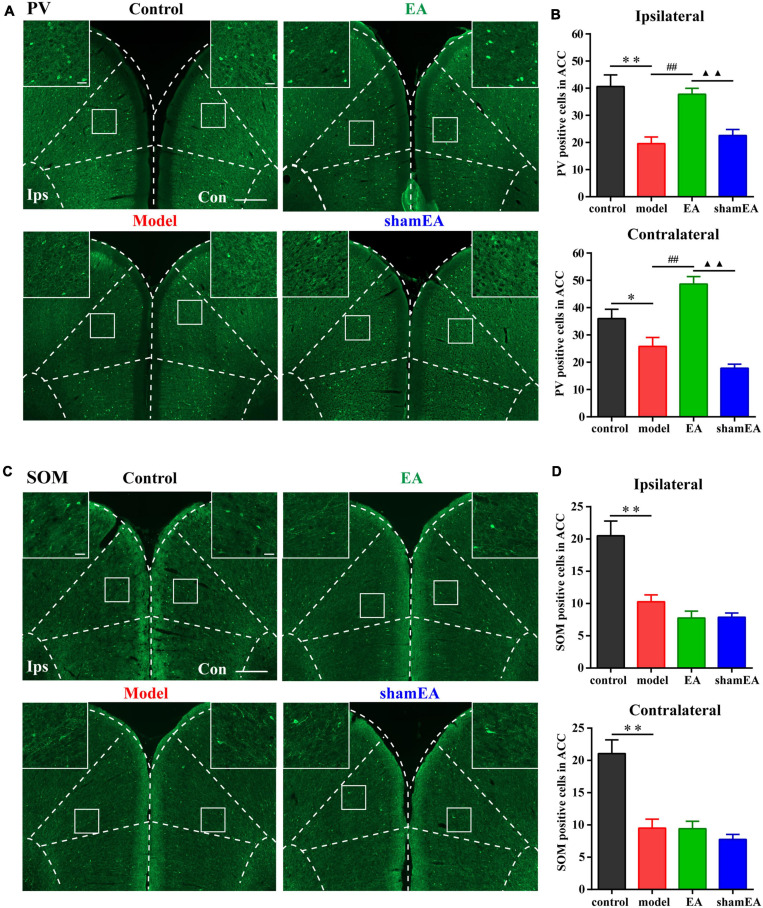
Electroacupuncture (EA) treatment increased the number of PV interneurons in the chronic inflammatory pain model. **(A)** Representative images of PV-positive cells in the bilateral ACC in the different groups of rats. **(B)** Quantification of the immunofluorescence results for PV-positive cells in the ipsilateral and contralateral ACC in the different groups (whole figure scale bars: 500 μm; local figure scale bars: 50 μm). **(C)** Representative images of SOM-positive cells in the bilateral ACC in the different groups of rats. **(D)** Quantification of the immunofluorescence results for SOM-positive cells in the ipsilateral and contralateral ACC in the different groups (whole figure scale bars: 500 μm; local figure scale bars: 50 μm). All data represent the mean ± SEM, *n* = 3–4/group. ^∗^*P* < 0.05 and ^∗∗^*P* < 0.01, control group vs. model group; ^##^*P* < 0.01, EA group vs. model group; ^▲▲^*P* < 0.01, EA group vs. shamEA group.

Since EA treatment and activation of PV interneurons in the ACC were shown to have similar effects on alleviating chronic inflammatory pain and pain-related anxiety-like behaviors, the question was whether EA was associated with PV interneurons? To address this question, we detected the number of PV-positive cells in the bilateral ACC after the EA intervention. The number of PV-positive cells in the bilateral ACC were significantly lower in the model group than those in the control group (*P* < 0.05; Figures 6A,B), and EA treatment increased the reduction in PV-positive cell number in the ACC due to the CFA injection (*P* < 0.01; Figures 6A,B). There was no significant difference between the shamEA group and the model group (*P* > 0.05; Figures 6A,B).

In addition, we also detected the number of SOM-positive cells in bilateral ACC after EA intervention. We found that EA treatment had no effect on the number of SOM-positive cells in the bilateral ACC (*P* > 0.05; Figures 6C,D).

### Chemogenetic Inhibition of PV Interneurons in the ACC Reversed the Effects of EA

**FIGURE 7:**
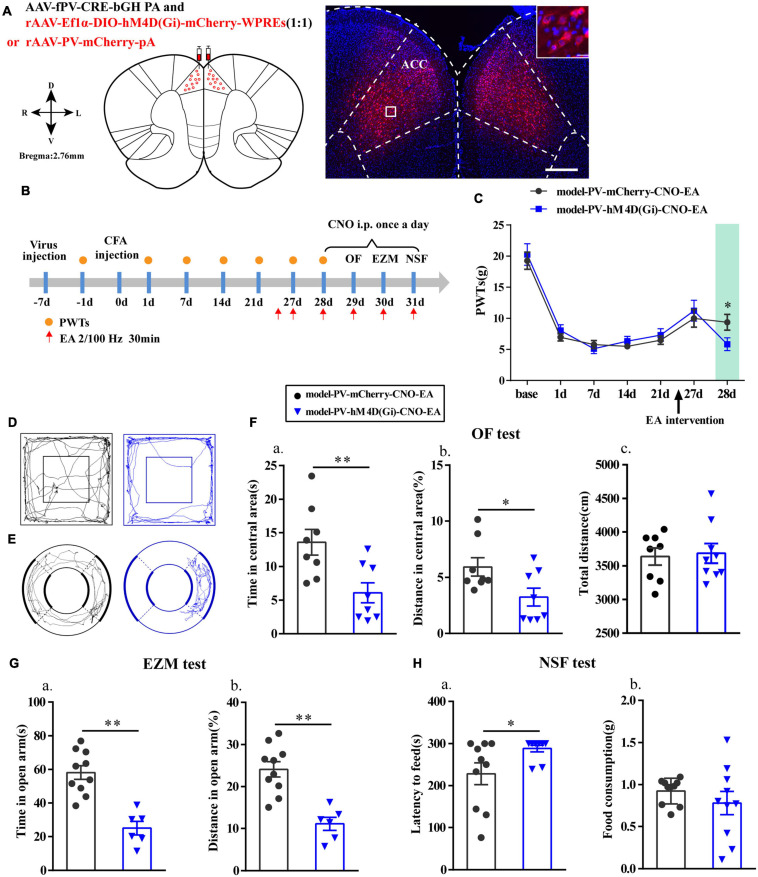
Chemogenetic inhibition of PV interneurons in the ACC reversed the effects of EA. **(A)** A representative figure showing expression of mCherry in the bilateral ACC (whole figure scale bars: 500 μm; local figure scale bars: 20 μm). **(B)** A schematic of the experimental design. **(C)** PWTs changes of chemogenetic inhibition of PV interneurons in the ACC (*n* = 12 in the model-PV-mCherry-CNO-EA group; *n* = 11 in the model-PV-hM4D(Gi)-CNO-EA group). **(D)** The trajectories of rats in the OF. **(E)** The trajectories of rats in the EZM. **(F)** Quantification of behavioral parameters in OF test (*n* = 8 in the model-PV-mCherry-CNO-EA group; *n* = 8 in the model-PV-hM4D(Gi)-CNO-EA group). **(a)** The time in the central zone, **(b)** the percentage of distance in the central zone, **(c)** and the total distance traveled throughout the arena. **(G)** Quantification of behavioral parameters in EZM test (*n* = 10 in the model-PV-mCherry-CNO-EA group; *n* = 6 in the model-PV-hM4D(Gi)-CNO-EA group). **(a)** The time in the open arm, **(b)** the percentage of distance in the open arm. **(H)** Quantification of behavioral parameters in NSF test (*n* = 10 in the model-PV-mCherry-CNO-EA group; *n* = 10 in the model-PV-hM4D(Gi)-CNO-EA group). **(a)** The time of latency to feed, **(b)** and the food consumption. All data represent the mean ± SEM. ^∗^*P* < 0.05, ^∗∗^*P* < 0.01, compared to the model-PV-mCherry-CNO-EA group.

Furthermore, we specifically inhibited PV interneurons in the ACC to assess alterations in the analgesic and antianxiety effects of EA. The location of the virus injection was showed in Figure 7A, and we found that cells with mCherry signals could be observed in the ACC. The flow chart of the experimental design is shown in Figure 7B. The PWTs in the two groups significantly decreased from 1 to 21 days after CFA injection (*P* < 0.01; Figure 7C). As before, the EA intervention was started on day 26 after CFA injection. Not surprisingly, the PWTs in the two groups of rats significantly increased after the EA intervention. However, the PWTs in the model-PV-hM4D(Gi)-CNO-EA group significantly decreased compared with those in the model-PV-mCherry-CNO-EA group after CNO administration (*P* < 0.05; Figure 7C).

Moreover, after intraperitoneal injection of CNO in rats, time in central area and percentage of distance in central area in the OF test, the time in open arm and the percentage of distance in open arm in EZM test were significantly reduced (*P* < 0.05; Figures 7D–G), and the feeding latency in the NSF test were significantly increased in the model-PV-hM4D(Gi)-CNO-EA group relative to those in the model-PV-mCherry-CNO-EA group (*P* < 0.05; Figure 7H). As expected, the hM4D(Gi) manipulation did not affect locomotor activity. These results showed that chemogenetic inhibition of PV interneurons reversed the effects of EA.

## Discussion

**FIGURE 8:**
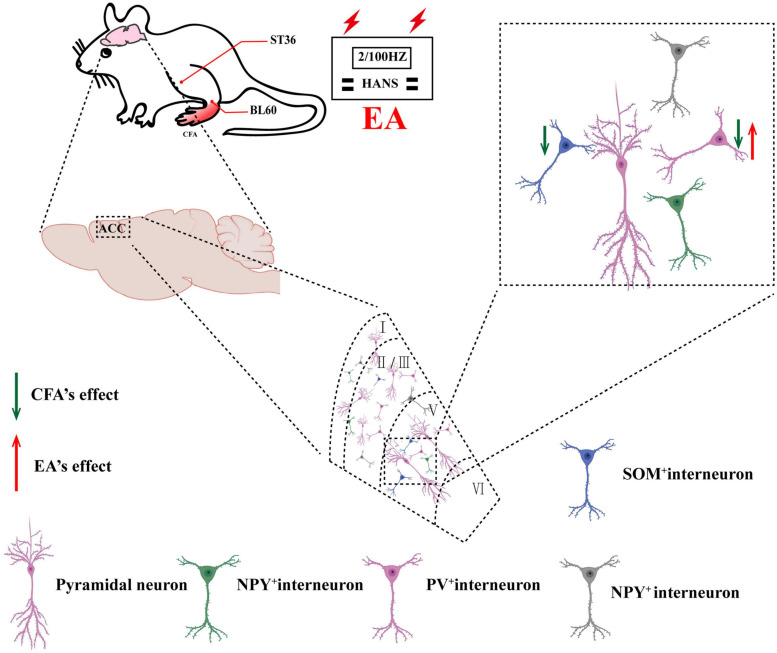
Schematic of EA ameliorates chronic inflammatory pain-related anxiety. EA’s effects on chronic inflammatory pain and pain-related anxiety may be mediated by improving the excitability of PV interneurons.

In this study, we demonstrated that activation of PV but not SOM interneurons in the ACC alleviated pain sensation and pain-induced anxiety in rats with chronic inflammatory pain. In addition, EA had analgesic and antianxiety effects and increased the expression of PV-positive cells but not SOM-positive cells. Furthermore, inhibition of PV interneurons in the ACC reversed the analgesic and antianxiety effects of EA. Therefore, we determined that EA ameliorated chronic inflammatory pain-related anxiety by upregulating the function of PV interneurons (Figure 8).

This series of discoveries provides compelling evidence regarding the high comorbidity between chronic inflammatory pain and anxiety, and this high comorbidity greatly affects people’s physical and mental health and quality of life (Parent et al., 2012; Arango-Davila and Rincon-Hoyos, 2018). Many researchers have suggested that chronic inflammatory pain is mediated by central sensitization and neuroplasticity (Latremoliere and Woolf, 2009; Walker et al., 2014). Previous evidence suggested that central sensitization can be caused by peripheral neuroinflammation characterized by the glial cells activation (Ji et al., 2014; Ji et al., 2019). Moreover, microglia and astrocytes activation will release various proinflammatory cytokines (e.g., IL-1β, TNF-α) and chemokines (e.g., CCL2, CXCL5), which can affect the excitatory (facilitation) and inhibitory synapses (dis-inhibition) (Ji et al., 2018; Latremoliere and Woolf, 2009). Human clinical studies have found that multiple areas of the human brain are activated in response to nociceptive stimuli, such as the ACC, prefrontal cortex (PFC), primary somatosensory cortex (S1), and thalamus (Bushnell et al., 2013; Malfliet et al., 2017). Among them, the ACC is thought to play a critical role in pain-induced affective responses, particularly pain-related unpleasant and aversive sensations. A previous study reported that cingulotomy mainly blocks emotions associated with pain (Wilkinson et al., 1999). Another animal study showed that injection of an excitatory glutamate antagonist into the ACC prevented formalin-induced positional aversion without relieving formalin-induced acute inflammatory pain (Johansen and Fields, 2004). In addition, our previous study found that inhibiting glutamatergic neurons in the ACC-thalamic neural circuity can interfere with pain-induced anxiety-related emotions (Shen et al., 2020). There is no doubt that all of this evidence suggests that hyperexcitability of the ACC is involved in pain and pain-related negative emotions.

Activity of ACC neurons is necessary and sufficient for the coding and processing of pain and the accompanying emotional pain, and the balance between neural excitation and inhibition is necessary for the normal function of the ACC (Gong et al., 2010; Zugaib et al., 2014). In chronic pain clinical practice and basic research, changes in neurotransmitters in the central system have been found, which was caused by the dysregulation of GABAergic, glutamatergic, dopaminergic and opioidergic mechanisms (Mhalla et al., 2010; Bannister and Dickenson, 2020; Yang et al., 2020). Dysfunction of GABAergic interneurons, as a major inhibitory system in the central nervous system, was reported to be closely related to the pathogenesis of anxiety-like behavior and chronic pain (Möhler, 2012; Lau and Vaughan, 2014). We focused on the function of GABAergic interneurons in ACC and curious about whether they were altered and involved in the generation of chronic pain-related anxiety in the persistent inflammatory pain rats. PV and SOM interneurons are the two major subtypes of GABAergic interneurons. Our results showed that chronic inflammatory pain induced by CFA led to a loss of bilateral PV- and SOM-positive cells in the ACC. Reductions in PV-positive cells have also been observed in several disease pathologies, including Alzheimer’s disease (Verret et al., 2012), schizophrenia (Marissal et al., 2018), fear learning (Çaliskan et al., 2016), cognitive deficits (Murray et al., 2015), autism (Lauber et al., 2016), and depression (Zhou et al., 2015). It was also found that PV-positive cells were decreased in a neuropathic pain mouse model (Shiers et al., 2018). Optogenetic activation or inhibition of PV interneurons in neuropathic mice causes an increase or decrease, respectively, in mechanical hypersensitivity (Zhang et al., 2015). SOM interneurons in the trigeminal subnucleus caudalis have been implicated in the processing of orofacial pain (Yin et al., 2009). To further assess the role of PV and SOM interneurons in chronic inflammatory pain and pain-related anxiety, we used chemogenetic methods to activate PV and SOM interneurons. Interestingly, anxiety-like behaviors and pain in these rats with chronic inflammatory pain were obviously alleviated by activating PV interneurons. However, chemogenetic activation of SOM interneurons did not have any effect on pain and pain-related anxiety. It had been shown that local interneurons of PV and SOM played different and coordinated functions in orchestrating neuronal oscillations (Chen et al., 2017). A recent study also demonstrated that ventromedial and mediodorsal thalamus interneurons participated in PFC different inhibitory networks by targeting either PV or SOM interneurons (Anastasiades et al., 2021). As reported that PV interneurons in the nucleus accumbens shell (sNAc) exhibited high excitability in anxiety mouse, and activation of SOM interneurons afferents from the anterior dorsal bed nuclei of stria terminalis affected PV interneurons and reduced anxiety-like responses (Xiao et al., 2020). Another a research indicated that type I corticotropin-releasing factor receptors in the BLA regulated fear and anxiety-like behaviors, which were mainly distributed in PV interneurons but less expressed in SOM interneurons (Calakos et al., 2017). Ji et al. (2020) demonstrated a selective reduction in PV but not SOM expression in the medial prefrontal cortex (mPFC) in a lipopolysaccharide (LPS)-induced neuroinflammation model, and overinhibition mediated by PV interneurons plays a distinct role in LPS-induced depression-like behavior. It was previously found that chemogenetic suppression of PV interneurons in the amygdala of naive mice can induce anxiety-like behavior (Luo et al., 2020). Studies have found that activation of PV interneurons but not SOM interneurons alleviated mechanical hypersensitivity in rats with chronic inflammatory pain (Kang et al., 2015). Our results appear to be consistent with these findings: activation of PV interneurons ameliorated chronic inflammatory pain and pain related anxiety. Interesting, SOM positive cells in bilateral ACC were decreased, however, chemogenetic activation of SOM interneurons did not have any effect on pain and pain-related anxiety. We speculated that declining SOM positive cells were not involved in chronic inflammatory pain and pain related anxiety. It is indicated that PV interneurons but not SOM interneurons were involved in chronic inflammatory pain and pain-related anxiety.

Electroacupuncture has been recognized in many countries as a kind of complementary and alternative medicine that can treat a variety of diseases (Han, 2004). Many acupoints were commonly selected for treatment of anxiety neurosis (Table 1). Some studies had confirmed that ST36 and BL60 have good analgesic and anti-inflammatory effects (Chang et al., 2014; Wang et al., 2017). The acupoints of ST36 and BL60 were generally used in our previous research and had been identified to be effective in chronic inflammatory pain (Xiang et al., 2019) and pain-related anxiety (Du et al., 2017). Therefore, we choose the acupoints of ST36 and BL60 in this study. As expected, repeated EA treatments obviously alleviated chronic inflammatory pain and pain-induced anxiety-like behaviors in OF, EZM, and NSF tests. Previous studies have mainly focused on mechanisms related to the sensory dimension of pain, such as EA alleviated the chronic pain through peripheral purinergic signaling, cytokines, opioid, cannabinoid, adenosine, transient receptor potential channels, etc. (Trento et al., 2021; Zhang et al., 2014; Lv et al., 2021), but attention has begun to turn to mechanisms related to the emotional dimension of pain in recent years (Lumley et al., 2011). Several studies have investigated the potential mechanisms by which EA prevents anxiety-like behavior. A study had found that adenosine released by acupuncture can reduce the input to ACC in response to nociceptive stimulation (Goldman et al., 2010). Recent study showed that EA alleviated pain and pain-related anxiety in rats with chronic inflammation by increasing the expression of NPS/NPSR system in the ACC (Du et al., 2020). Another study found that EA suppressed the anxiodepressive-like behavior of rats with neuropathic pain by restoring hippocampal NR1 phosphorylation (Li et al., 2014). This study demonstrated that PV interneurons participated in the regulation of chronic inflammatory pain and pain-induced anxiety. However, there was still no direct evidence on whether the effects of the EA intervention on chronic inflammatory pain and pain-induced anxiety were realized through the PV subtype of GABAergic interneurons. In this study, immunofluorescence results showed that EA treatment increased the number of PV-positive cells in the bilateral ACC, but did not change the number of SOM-positive cells. To further elucidate the underlying mechanisms of EA on chronic inflammatory pain and pain-related anxiety-like behavior, we combined EA treatment with the chemogenetic methods for inhibition of PV interneurons. The results showed that rats in the model-PV-hM4D(Gi)-CNO-EA group displayed higher anxiety-like behaviors in the OF, EZM, and NSF tests and a lower pain withdraw thresholds after CNO intraperitoneal injection than rats in the model-PV-mCherry-CNO-EA group. These results suggested that EA’s effects on chronic inflammatory pain and pain-related anxiety may be mediated by activation of PV interneurons.

**TABLE 1:** Acupoints with anti-anxiety effect in acupuncture therapeutics.

**Acupoints**	**Disease-related anxiety**	**References**
HT7	Alcohol dependence	Chang et al., 2019
GB34, ST36	Parkinson	Jang et al., 2020
ST36	Post-traumatic stress	Liu et al., 2019
GV20, GV29	Cocaine dependence	Nie et al., 2020
GB34, Du20	Chronic unpredictable stress	Yue N. et al., 2018
ST36, SP6	Chronic inflammatory pain	Shen et al., 2020
ST36, BL60		Du et al., 2020
GB30	Chronic neuropathic pain	Shao et al., 2015
ST36, GB34		Zhang et al., 2021

## Conclusion

Electroacupuncture ameliorates chronic inflammatory pain and pain-related anxiety by upregulating PV but not SOM interneurons in the ACC.

## Data Availability Statement

The original contributions presented in the study are included in the article/supplementary material, further inquiries can be directed to the corresponding author/s.

## Ethics Statement

The animal study was reviewed and approved by the Animal Protection Agency used committee and the Animal Ethics Committee of Zhejiang University of Chinese Medicine.

## Author Contributions

FS and JFF performed the data analysis and wrote the manuscript. MQ, SW, and DX performed the experiments. XS and XH revised the manuscript. JD and JQF designed the experiment. All authors contributed to the manuscript and approved the publication of the final manuscript.

## Conflict of Interest

The authors declare that the research was conducted in the absence of any commercial or financial relationships that could be construed as a potential conflict of interest.
